# Management of retained trans-anal foreign body: a case report

**DOI:** 10.11604/pamj.2022.42.105.29651

**Published:** 2022-06-08

**Authors:** Edward Asumanu, Clement Nii Akwei Akwei, Abdullai Saeed, Jermaine Addai

**Affiliations:** 1 Department of Surgical Division, Military Hospital, Accra, Ghana,; 2 Department of Anaesthesia Division, Military Hospital, Accra, Ghana,; 3 Perioperative Nursing Division, Military Hospital Institution, Accra, Ghana

**Keywords:** Retained foreign body, anal sphincter, body spray can, case report

## Abstract

Management of retained trans-anal foreign bodies is yet to be standardized. We present a case of how a retained trans-anal colonic body spray can affect the anal sphincter and its effect on management. A male adult presented with a retained trans-anal body spray can in the colon and a weakened anal sphincter. A plain abdominal X-ray and colonoscopy confirmed the diagnosis. After a failed endoscopic removal, a non-operative manual removal was done. Sphincter tone improved 3 days later. The article describes anal sphincter weakness, patient categorization, and non-operative management of the retained colonic foreign body.

## Introduction

Retained trans-anal foreign bodies (RTFB) are objects inserted through the anal canal and have become entrapped in the rectal or colonic lumen. It occurs commonly from sexual use, and it is a surgical condition that has seen increasing reports in the past decade [1-3]. Retained trans-anal foreign bodies present to emergency departments and requires a high index of suspicion in the diagnosis [1]. These foreign bodies do have an impact on the tone of the anal sphincter by their mechanical stretch forces and lead to varying degrees of weakness and incontinence. Bottles impulse, body spray cans, fruits, and vegetables are some examples of retained foreign bodies [2,4]. The commonest reported of these are household objects [3]. Most of these foreign bodies are located in the rectum. The foreign bodies that are retained in the colon present a management challenge. The shape and size of the object determine the degree of anal sphincter muscle weakness and their ultimate retained site. The body spray can has a unique shape that encourages its use and determines the effect it produces on the anal sphincter tone and its colonic location. We present a case of how a retained body spray can following trans-anal insertion, affect anal sphincter tone and a management approach.

## Patient and observation

**Patient information:** a 60-year-old male expatriate reported to the emergency surgical unit of our hospital with a persistent mucoid discharge per rectum. The symptom occurred 5 days after self-insertion of an empty body spray can. The foreign body was inserted during sexual experimentation. He had noted the passage of mucoid feces with incontinence. There was no bleeding per rectum or pain. He was actively working and lived alone. There was no significant psychosocial past medical history. He had first reported to a private general practice facility where a rectal examination failed to identify any object in the rectum and was therefore referred to a tertiary facility.

**Clinical findings:** his general examination was unremarkable except for mild tenderness along the left iliac fossa. There was no palpable mass on the abdominal examination. Rectal examination confirmed weakness of the anal sphincter with clear mucoid discharge but the foreign body was not palpable.

**Figure 1:**
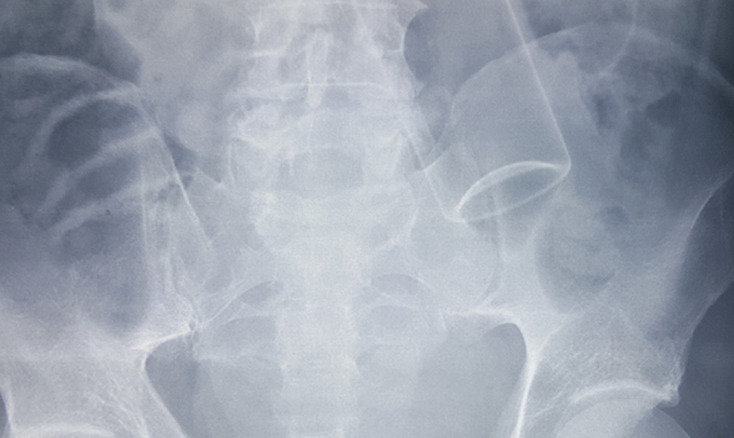
pelvic X-ray showing a left colon foreign body

**Diagnostic assessment:** a pelvic X-ray done showed a cylindrical radio-opaque foreign body above the iliac crest Figure 1. Full blood count and blood urea and electrolytes were normal. An emergency flexible colonoscopy showed the retained foreign body 20cm from the anal verge, with a visible external description of the object as a “Body spray” and mucosal ulceration around the area of impaction.

**Therapeutic interventions:** removal of the body spray can was unsuccessful during the flexible endoscopic procedure, which was easy to conduct. The colonoscope was unable to maneuver the spray can distally from its position. The patient was therefore prepared for examination and removal under general anesthesia with muscle relaxation. During the procedure, the patient was placed in a reverse Trendelenburg position. The body spray can be manipulated into the rectum bimamually which allowed a successful removal through the anal canal.

**Outcome and follow up:** the sphincter tone returned to normal three days after the removal of the body spray can. The patient was able to move his bowel normally and did not experience any abdominal pain or fever. He was discharged on postoperative day 5 and there were no observed complications 6 months later.

## Discussion

There is an increasing incidence of retained trans-anal foreign bodies noticeable in men [5,6]. However, there are no standard medical protocols for the management of these patients with retained foreign bodies [7]. Body spray can as a RTFB has been reported in many cases [2,8-10]. The case presented shows anal sphincter weakness that occurs with retained body spray can, but improves within days after removal of the foreign body. This suggests that the mechanism is temporary and likely due to the stretch of the anal sphincter muscle during transit by the object. The mechanism of the anal weakness is attributable to the shape of the object. The body spray can has a cylindrical body with a conical top and a smooth outer surface covering. The conical top shaped like a proctoscopy facilitates its insertion while the cylindrical body causes a persistent anal dilatation resulting in the temporary sphincter weakness. The characteristic shape may be the reason for its choice as a preferred object for autoerotism in men. The demographics of the case highlight a pattern and a challenge in management. The use of rectal sex toys are likely to increase with the change in sexual orientation and the desire for autoerotic [11]. It is a cross-cultural phenomenon, diagnosed in the emergency room but difficult to manage in the emergency room [8]. The practice is not socially acceptable in most African countries and the stigmatization will result in delayed presentation and management. The presence of anal sphincter weakness and incontinence is distressing for the patient and maybe a confusing presentation for the clinician. Anal sphincter tone is important in the maintenance of continence to solid and gas. The successful restoration of continence, in the case, presented, after removal of the body spray can indicate the benign nature of the condition. Anal sphincter weakness when present is an indicator of urgent intervention.

The characteristic tubular shape on plain pelvic or abdominal X-rays is a useful feature in the diagnosis of retained body spray cans. Plain abdominal X-rays will be a useful initial diagnostic investigation. Others reported that retained foreign bodies have characteristic appearances [1,12]. The patulous anal canal makes rectal examination as well as flexible endoscopy relatively easy. In our reported case, the flexible endoscopy depicted the white reflection of the base and was able to visualize the outer label, which was diagnostic of the type of object and location. Management of these cases requires an understanding of the challenges in diagnosis and retrieval of the foreign body [2,12,13]. The condition of the retained foreign body has become more common in recent times, with different approaches to a removal proposed [14]. The features of the body spray can make it difficult to remove endoscopically as was evident in this case where endoscopic removal failed. Early referral and examination under anesthesia have been proposed because emergency room procedures are associated with a high failure rate and often advance the objects high into the recto-sigmoid region [12]. A simplified management approach takes advantage of the weakened sphincter. The presence of anal sphincter weakness permits a double finger rectal examination. This allows for categorizing the retained colorectal foreign bodies for the purposes of removal. The ability to palpate the full circumference of the foreign body categorizes it as palpable. The uncomplicated retained foreign bodies should be easily grouped by rectal examination into; a) palpable retained trans-rectal foreign body and b) non-palpable retained trans-rectal foreign body. Palpable retained trans-rectal foreign bodies can then be removed at the emergency room under sedation. Non-palpable retained trans-rectal foreign bodies will often require relaxation under anesthesia in a lithotomy and reverse Trendenlenburg position. The addition of muscle relaxation makes bimanual manipulation and removal more successful. Post removal, patients should have proctosigmoidoscopy for any mucosal ulceration and must be observed for any perforation in 2-3 days. Plain abdominal X-rays or direct observation are useful alternatives for patient follow-up.

**Ethics:** the proposal was approved with IRB Number 37MH-IRB/CRS/IPN/495/2021

## Conclusion

Anal sphincter tone weakness that occurs with the retained trans-anal foreign body is temporary. The transient sphincter weakness can be used in patient categorization for the targeted management. Manual removal under anesthesia with muscle relaxation is a recommended approach to the management of non-palpable retained trans-anal foreign body with sphincter weakness.
